# On pharmacological neuroenhancement as part of the new neurorights' pioneering legislation in Chile: a perspective

**DOI:** 10.3389/fpsyg.2023.1177720

**Published:** 2023-07-18

**Authors:** María Isabel Cornejo-Plaza, Chiara Saracini

**Affiliations:** ^1^Neurometa Research Center in Neurorights, Neuroethics, Metaverse, Behavioural Economics and Artificial Intelligence, School of Law, Universidad Autónoma de Chile, Santiago, Chile; ^2^The Neuropsychology and Cognitive Neurosciences Research Center (CINPSI Neurocog), Faculty of Health Sciences, Universidad Católica del Maule, Talca, Chile

**Keywords:** Chile's neurorights constitutional reform, neurorights, neuroenhancement, ludic neurotechnology, distributive justice, smart drugs

## Abstract

The so-called neurorights are emerging human rights, or rather reconfigurations of already existing human rights, seeking to address the impact of the possible misuse of neurotechnologies, which have the potential to become more invasive and harmful in the future if not regulated. The aim of specifying neurorights is to protect the dignity and autonomy of the individual in the face of neurotechnological advances. Recently, Chile proposed a Constitutional reform inspired by the neurorights, opening a debate. One of the proposed neurorights is fair and equitable access to cognitive enhancement, which will be the specific object of this perspective article. Starting from the legal proposal, we analyse and discuss some perspectives on cognitive enhancement, or “neuroenhancement”, which could be considered as part of enhancement neurotechnologies, pointing out that pharmacological enhancers, or “smart drugs”, might be considered as part of these enhancers. We present a classification of the different types of cognitive enhancements as it has been proposed in the literature, into which pharmacological cognitive enhancement can be included, concluding that there is currently no agreement amongst scholars and lawyers about the ethical consideration of pharmacological cognitive enhancement. We therefore argue that it is necessary for the legislator to explicitly address the issue in the proposed regulations, in order to take a clear position on the topic, as it has been done in the United Kingdom, where the pharmacological neuroenhancers have been explicitly excluded from the regulation. If pharmacological neuroenhancers are going to be considered neurotechnologies, then new law proposals should seek harmonization with the already existing legislation regulating pharmacological health and consumer rights (both globally, taking into account international drug laws, and locally, according to each country's internal regulations) and of course, with the whole system of fundamental rights. Finally, we briefly discuss the ethical problem of equitable access to this new type of neurotechnologies (as part of the neurorights) and leave the debate open for new insights from the scientific community on the possible consequences of including (or not) pharmacological neuroenhancers as neurotechnologies for cognitive enhancement in the framework of the ethical and legal debate.

## 1. Introduction

Neurorights are new human rights, or rather reconfigurations of some emerging rights, proposed by some scientists in an attempt to prevent possible bioethical issues that may arise from the rapid scientific and technological advances in the field of neuroscience, which are bringing us closer every day to unraveling the mysteries of the last frontier of science: the brain. Rafael Yuste, a neuroscientist from the B.R.A.I.N. project (Kandel et al., 2013), began discussing this topic with many colleagues in the Morningside Group, and these conversations coalesced into the NeuroRights Initiative (NRI), a renewed effort by scientists and entrepreneurs to raise awareness about the human rights and ethical implications of neurotechnology. The NRI Manifesto proposes four fundamental rights for all human beings in the near future: Privacy and consent; Agency and identity; Augmentation; Prejudice (Yuste et al., 2017).

Among scholars, the idea that our future societies would benefit from new human rights called neurorights had not been put forward only by Yuste et al. (2017): in the same year, Ienca and Andorno (2017) also proposed four new neurorights: Cognitive Liberty; Mental Privacy; Mental Integrity and Psychological Continuity. The main differences between this other proposal and the set of rights proposed by Yuste and colleagues are that Ienca and Andorno's proposal does not consider the equitable right to cognitive augmentation, nor the right to protect humans from the biases of algorithms or automated decision-making processes, although they recognize that current and future scenarios of cognitive neuroenhancement are already occurring, and the threat of malicious use of data generated by brain activity might be more than a chance.

Cognitive augmentation or neuroenhancement is one of the emerging human rights that has sparked a debate (Borbón and Borbón, 2021; Bublitz, 2022; Fins, 2022; Rainey, 2023) for moral and legal reasons, beyond a certain consensus on the need to regulate cognitive enhancement neurotechnologies (Ienca, 2021a; RHC, 2022). From a therapeutic point of view, there are no moral issues. Rather, the problem arises when healthy people wish to enhance their cognitive capacities, because cognitive enhancement could grant unfair advantages to those better placed in society, constituting a gap either by exacerbating existing inequalities or by opening the way to new inequalities that are rooted in the fact that it is not possible for everyone to have access to safe and sustainable neuroenhancement. But this “right” has complicated ethical boundaries, as we will discuss later.

Chile has been the first country to propose a constitutional reform on the protection of neurorights, modifying article 19, N° 1, the last paragraph of the Fundamental Chart, on the protection of integrity and mental indemnity in relation to the development of neurotechnologies. In addition to this law, a bill (contained in Bulletin 13.828-19), colloquially known as “neurorights” or “regulation of neurotechnologies”, is currently being processed. This bill, which complements the constitutional reform of neurorights recently approved unanimously by the Chilean Senate (December 2021), is currently under discussion in the Chamber of Deputies. Chile is currently discussing for the second time a draft of a new Political Constitution of the Republic, after the rejection of the first text submitted to the plebiscite on 4 September 2022, which did not include neurorights. However, in this new constituent process, the Commission of Experts has proposed to maintain exactly the same content of the constitutional reform on neurorights approved in 2021, which will be submitted to the Constitutional Council for a plebiscite during the course of 2023.

The theoretical and empirical foundations inspiring and endorsing the Chilean proposal are rooted in the reflections that originated in the context of the series of conferences called “Congreso Futuro” (or “Future Congress” in English, organized by the Senate Committee called “Comisión Desafíos del Futuro, Ciencia, Tecnología e Innovación” del Senado de la República de Chile between 2018 and 2022), where Yuste presented his view on the need for neurorights in future societies. His thoughts have been influential in the drafting of both the neurorights proposal in the Chilean constitutional reform and the bill on the regulation of neurotechnologies. In fact, the proposal for constitutional reform has been largely discussed for two years in mixed committees, in which Yuste participated, and it has been sanctioned on October 25, 2021, becoming Law No. 21.383 that “Amends the Fundamental Charter” to “establish scientific and technological development at the service of people”. This law consists of a single article that modifies paragraph 1 of article 19 of the Political Constitution of the Republic of Chile as follows:

*[...] “Scientific and technological development shall be at the service of people and shall be carried out with respect for life and physical and psychological integrity. The law shall regulate the requirements, conditions and restrictions for its use on people, and shall especially protect brain activity, as well as the information coming from it” [...]*.

It is clear that there is a need to fill a legal gap on these upcoming issues, as technology and science are moving so much faster than 10 or 20 years ago in terms of the normative updating process. But what is the best way to proceed along this unexplored path? Some authors stress the importance of taking a step-by-step approach, first improving existing regulations before proposing new legislation; indeed, at this initial stage, given the diversity and variety of Human Enhancement Technologies (HET; as defined, for example, by Siemaszko et al., 2020), and the actual low level of institutionalization of this newborn field, a regulatory approach that aims to address all relevant issues in one legal instrument at a time might be a worse strategy than pursuing a gradual building of understanding and consensus through a series of legal instruments. This consensus clearly hasn't been reached yet, and the current legislative proposal in Chile did not follow an incremental strategy. Indeed, neurorights as a legal proposal has been criticized (see for example Borbón Rodríguez et al., 2020; López-Silva and Madrid, 2021; Ruiz et al., 2021; Zúñiga-Fajuri et al., 2021; Bublitz, 2022; Fins, 2022; Hertz, 2023; Rainey, 2023).

The Chilean law proposal prompted various reactions also in Chilean society from the political, legal, professional and academic sides, raising questions such as: is it useful to have some “neuro” rights? Is data derived from brain activity really special enough to need a law specifically protecting it (wouldn't it be enough to create a good law for the protection of personal and biological data)? If a country strictly regulates the use of neurotechnologies, could this limit scientific and technological progress to improve people's lives? The debate is still open, and there are many active authors providing a deeper insight into all the different positions (for an updated review on the topic, see Ligthart et al., 2023).

Nevertheless, the pioneering proposal of the Chilean neurorights bill raises some concerns about another issue that we believe has not received enough attention, and that is related to the inclusion (or not) of pharmacological neuroenhancers in the definition of the neurotechnologies it will regulate (and if included, how to deal with equitable access to this type of enhancement). It can be said that Yuste and Goering's proposal has been included in the discussions of both Chilean neurorights projects (the reform of the Political Constitution of the Republic of Chile and the bill for the regulation of neurotechnologies). However, the right to cognitive enhancement and its equitable distribution does not have a specific article enshrining this right in the bill that is currently being drafted (Regulation of Neurotechnologies).

Moreover, the Chilean draft law contains a definition of neurotechnologies that neither excludes nor explicitly includes all those pharmacological cognitive enhancements or “neuroenhancing drugs” (Cornejo Plaza, 2021a,c). The official definition of “neurotechnologies” in Article 3 of Bulletin 13.828-19 is as follows “*A set of devices, methods or instruments that allow a connection with the nervous system, the recording of brain activity and the information coming from it”* (see here for the original version of the bill). This is a broad definition that could include any new technology designed to interface with and enhance human mental functioning by altering brain function, either to restore or overcome a lost or failing function (e.g., in clinical patients), or simply to improve a person's cognitive abilities or performance. The purpose of enhancement is generally to improve subjective wellbeing and quality of life. However, it cannot be considered a therapeutic treatment in the strict sense, as, for example, aesthetic surgery, doping in sports and the use of anti-aging drugs can also be considered enhancements to some extent (Brukamp and Gross, 2012). Among the many neurotechnologies for cognitive enhancement (we will discuss some classifications in the next sections), the most accessible and easy-to-use to the public are those available in pharmacological form.

Neuropharmacology aims to study and develop synthetic drugs able to cause behavioral changes altering mental or brain functions due to their chemical action on the nervous system for medical purposes (Mohamed, 2017). Some of these psychoactive substances (and their properties) belong to the so-called “nootropics” (Giurgea, 1972) and affect the functioning of the central nervous system either by altering the concentration of neurotransmitters and other neurochemicals or by increasing the availability of oxygen in the brain; instead, from a structural point of view, a limited class of nootropics is able to directly stimulate cell growth or regeneration (see Ienca, 2018 for a categorisation). Besides their therapeutic use, the effects of certain drugs can be exploited in social contexts, providing pleasure or improving cognitive and/or emotional performance. Among these substances, for example, two of the most widely accepted from a legal and social point of view are alcohol and nicotine, but we can also mention stimulants such as caffeine (Nehlig, 2010) and guaraná (Haskell et al., 2007), and even glucose (Smith and Farah, 2011), all of which have been shown to improve cognition to some extent (Dresler et al., 2019). However, there are also a number of unregulated substances, generally used by restricted categories such as students, academics, surgeons and business people. These are defined as pharmacological neuroenhancers (PN; Lucke et al., 2011), which are also defined as pharmacological cognitive enhancers (PCE; Franke et al., 2011); here we use these two definitions interchangeably.

In fact, while “neuroenhancement” is generally referred to as the improvement or enhancement of cognitive abilities using medical devices for therapeutic purposes and subject to strict medical and ethical rules, the term has recently been associated with a different context. In some cases, the terms “PN” and “PCE” are also used to refer to the use of illicit or prescription drugs by healthy people for cognitive enhancement purposes (Franke et al., 2011; Dietz et al., 2018); an example is methylphenidate, which is usually prescribed for the treatment of ADHD, but has been found to improve certain cognitive performance (see Repantis et al., 2010 and Caviola and Faber, 2015 for reviews). This means that people who do not suffer from a diagnosed pathology take PN essentially as “smart drugs” to “cognitively dope” themselves (Lucke et al., 2011), using biomedical development for non-therapeutic purposes. Cognitive performance can be pushed above “normal” in order to cope with successful and competitive lifestyles with demanding jobs or high levels of decision stress or many hours of physiological arousal.

This “recreational” use of cognitive augmentation (“recreational” as opposed to strictly medical use) is a growing practice, particularly among university students, academics, doctors, surgeons, military pilots, athletes, managers, and businessmen, due to the high physical, mental and cognitive demands they face on a daily basis (see Fronda et al., 2018). Depending on a number of factors, such a practice could alter the functioning of the central nervous system and eventually lead to addiction (Volkow and Swanson, 2008; Mohamed, 2012) or other altered states such as increased heart rate and blood pressure, headaches, anxiety, dizziness and insomnia as side effects of methylphenidate and modafinil use (Repantis et al., 2010). These are medicines that are used off-label for non-therapeutic purposes. Most of them are of course regulated, sometimes very strictly, only in a therapeutic context. Efforts for a global regulatory framework are trying to address many of them (WHO, 2019), trying to define the appropriate use of medicines and a fair distribution among the population. The guidelines represent a soft-law approach that does not, of course, replace the objective laws of each country. Similar substances, such as amphetamines or psychotropics/nootropics, are regulated by national medical laws, as in the case of the FDA regulations in the US; however, in the case of Chile, the drug legislation does not provide for the non-therapeutic use of nootropics, i.e., neuroenhancement (Law 20.724, which amends the Health Code on the Regulation of Pharmacies and Medicines).

Of course, in most of the western countries, neuropharmaceuticals have a specific regulation. However, as many scholars consider these enhancers as a neurotechnology for cognitive augmentation, the Chilean legislation (or any legislation willing to regulate neurotechnologies or include neurorights in its legal structure in the future) should explicitly state whether it should be included in the regulation of neurorights or not. A good example of a clear position is the UK model regulation proposal (RHC, 2022), which excludes drugs from the regulation of neurotechnologies, without prejudice to the recognition that they are also neurotechnologies. Another solution could be to include the specific use of nootropics as enhancers in the regulation of drugs, but this could of course raise further questions.

The idea is to avoid producing a legal uncertainty, or a sort of legal loophole whether neuroenhancement should be regulated or whether the specific regulation should be revised (Bublitz, 2022). The proposal to legislate and regulate neurotechnologies in Chile, within the framework of Neurorights, has opened a discussion because the legislator seems to assume that the practice of neuroenhancement does not exist in Chile and therefore the sanitary statute, which prohibits the acquisition of nootropics for non-indicated uses, must be applied. However, ignoring the fact that the practice of neuroenhancement exists and is growing every day, despite the Sanitary Statute, implies that there is a wide market of neurotechnological devices that are not intended for therapeutic use, but for recreational use, and that fall outside the scope of the Sanitary Statute. The Chilean neurorights legislation does not specify whether any of the neurorights proposed by Yuste are excluded. Therefore, it could be considered that pharmacological enhancement is included in the neuroright to “equitable access to cognitive enhancement”, as the authors do not explicitly exclude it (Yuste et al., 2017). This legislative omission could lead to interpretations that consider pharmacological enhancement as a neuroright, as there is no differentiated status for it. This is important, because, as we will discuss later, it has ethical implications. We believe that in the case of nootropics, a specific regulation should be considered and discussed between the markets, politicians, scholars, scientists, civil societies, consumers, and most importantly, within a bioethical perspective.

## 2. Classification of neuroenhancements

With respect to the “general” enhancements, such as those already mentioned in the context of aesthetic surgery or the consumption of vitamins or dietary supplements, neuroenhancers are a specific class of improvements that have a direct or indirect effect on the Nervous System, and more specifically, on the Central Nervous System, being the ones involving the Peripheral System very few (Brukamp and Gross, 2012). In this sense, current and future neurotechnological developments have the potential to open the “Pandora's box” of seemingly infinite possibilities for interacting and manipulating the structure and functioning of our biological substrate which, in essence, enables the most intimate and private experience of “being oneself”. Based on technical resources, cognitive improvement can be genetic, pharmacological, or electromagnetic (Transcranial Magnetic Stimulation, Direct/Alternate Current Stimulation Ultrasound Stimulation, Deep Brain Stimulation), surgical (transplantation of neural prostheses, transplantation of intracranial cellular tissue, especially stem cells or embryonic cells), or optometric (light stimulation).

Increasing attention has been paid to external and non-invasive forms of brain stimulation, such as Transcranial Magnetic Stimulation (TMS) and Transcranial Direct/Alternate Cur-rent Stimulation (tD/ACS), which, in addition to their relevance for the treatment of conditions such as depression, ADHD, Parkinson's disease, schizophrenia and many others, show great potential for improving both mood and cognitive functions such as memory, mathematical ability and language learning (Erler and Forlini, 2020). It even seems to allow the reduction of “racial bias or propensity to aggression” (Harris, 2011; Douglas, 2013; Focquaert and Schermer, 2015). The most futuristic forms of cognitive enhancement are giving way to incredible possibilities, including neural implants directly connected to the Internet and machine-human hybridisation, allowing our abilities, including cognition, to benefit exponentially from artificial intelligence technology (Kurzweil, 2014).

Brain-computer interfaces (BCIs), on the other hand, have gained popular attention since Elon Musk presented his Neuralink experiment with a monkey playing Pong (Wakefield, 2021). However, the neurosciences had already made more or less the same attempt in the early 2000s (Donoghue, 2002; Tangermann et al., 2008). At that time, these incredible technologies were still *in nuce*. People like Jan Scheuermann (Collinger et al., 2013), Nathan Copeland (Flesher et al., 2016) and Dennis Degray (Corbyn, 2019) have chosen to have a BCI-controlling chip implanted directly in their head in order to be autonomous and independent despite their health conditions, but these cases did not reach the public like Musk's developments, because he has all the tools and knowledge to to capture the attention of consumers. After the monkey demonstration, Musk promised that he is going to use the same Neuralink technology to enhance human capabilities through microchip implants that would allow direct implementation of BCIs (Wakefield, 2021 citing the Neuralink blog post from Abril, 18, 2021), paving the way for a kind of cyborgisation of humans and attracting more attention than scientific experiments. In addition to the great potential of these devices for functional rehabilitation of clinical patients, the most debated and exciting use of these futuristic neurotechnologies is in the leisure sector. When the “patient” becomes a “client”, things change dramatically, as the focus is usually on profit and not just on the actual benefit to the person. This area of development is, in fact, the most unregulated and requires a concerted dialogue and reflection between different actors (scientific, medical, legal, and ethical).

In the scientific literature, some attempts have been made to classify enhancements according to characteristics, technologies, methods or affected functions (Farah et al., 2004; Brukamp and Gross, 2012; Dresler et al., 2019). These classifications imply different dimensions and modes of action, each emphasizing different aspects on which there is clearly no agreement at this stage. Nevertheless, they all focus on the different effects of enhancement on mood, emotions, social and moral behavior vs. cognition. For example, Brukamp and Gross (2012) consider six categories of neuroenhancement according to cognitive functions (Sensory perception; Motor action; Communication; Mood and emotions; Cognitive processes, such as attention, memory, decision-making; Social and moral behavior) and other four categories according to the used methods: Pharmacology; Interventions (surgical or minimally invasive); Non-invasive, external technology; and Invasive, internal technology.

**Figure 1:**
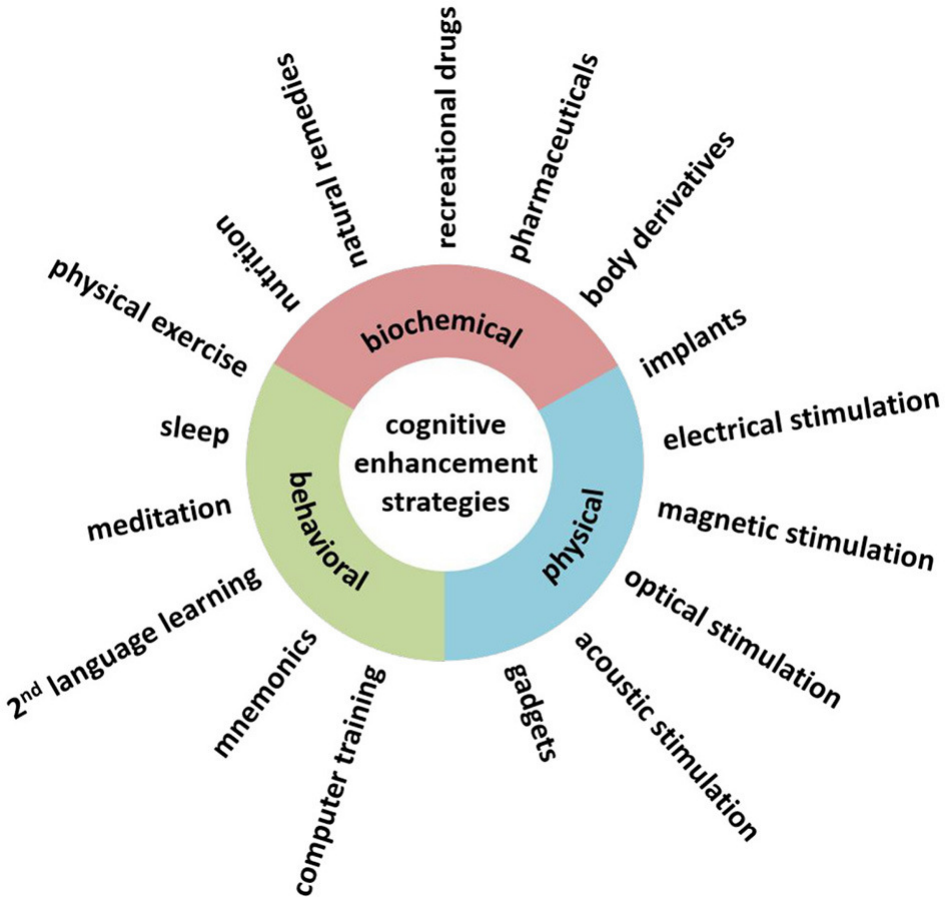
Cognitive enhancement interventions different in their mode of actions (Reproduced from Dresler et al., 2019, licensed under CC-BY-NC-ND).

Dresler et al. (2019) offer two classifications: one related to the dimensions of cognitive enhancement (mode of action, the targeted cognitive domain, personal factors, time scale, side effects, availability, and social acceptance) and one suggesting three different possible areas of intervention based on their mode of action (biochemical, physical, or behavioral); as a reference, see Figure 1, taken from the author's original paper.

The criminal lawyer Reinhard Merkel has developed a classification of the different devices for neuroenhancements from a legal perspective (Merkel, 2011). Within the general classification that he proposes, cognitive enhancement would be an “internal” type. Similarly to the aforementioned ones, his classification aims to differentiate improvements according to their goals or mental states, establishing three categories: i) the improvement of cognitive faculties, especially executive ones; ii) the improvement of emotional states: character traits, moods, social interests, etc.; and iii) the improvement of motivational states, including moral improvements, which have been used in relation to the prescription of neuropharmaceuticals to reduce aggressive impulses (Merkel, 2011).

Despite the different emphasis placed on each aspect of these classifications, they all agree on the fact that pharmacological neural enhancement is in some way part of these methods of enhancing “normal” human functioning, even if it doesn't constitute a “technology” in the usual sense of the term.

## 3. Pharmacological cognitive augmentation

Although scholar's interest in the topic of PCE (from a scientific, ethical and legal point of view) has increased since the 1990s (Whitehouse et al., 1997), the practice has existed and is documented for at least 80 years (Schleim and Quednow, 2018); if we include undocumented cultural and traditional practices such as exercise techniques and the consumption of natural extracts of neuroenhancing substances such as ginkgo biloba, ginseng, cocaine, guaranà, yerba mate, coffee, yaupon holly, and many others (Lloyd, 1911 cited in Ienca, 2018), these practices may have existed since ancient times. This has led to some criticism of the neuroenhancement debate itself, as Schleim and Quednow report in their argumentation; independent authors have referred to the topic itself as a “myth”, a “bubble” and a “phantom debate” (respectively, Quednow, 2010; Lucke et al., 2011; Zohny, 2015 cited in Schleim and Quednow, 2018). Nonetheless, they recognize that the issue requires careful debate and reflection in the scientific community, as agreement needs to be reached, at least operationally.

According to specialized literature, neuroenhancing drugs optimize cognitive and attentional performance, improve processing speed and accuracy, enhance attention and facilitate retention in learning processes (Repantis et al., 2010; Marraccini et al., 2016), creating comparative behavioral advantages in favor of those who make use of them. Although it could be considered a placebo effect by many opinionists, it is estimated that these substances can also have effects in the motivational or emotional domain (mood enhancement; Davis, 2013); affective enhancement also includes the socially accepted modification of personality through reward, including mood, motivation and pro-social behavior improvement, removal of unwanted or traumatic memories, and modulation of romantic relationships between people (De Jongh et al., 2008; Savulescu and Sandberg, 2008; Lavazza, 2019; Erler and Forlini, 2020). The specific effects of PCEs, according to De Jongh et al. (2008) and his research group, act on a variety of neurotransmitter systems that appear to be capable of improving: (a) cognition, specifically working memory, executive functioning (spatial planning ability), sustained attention and episodic memory; (b) state of mind, although to a lesser extent than cognition, also increasing “tolerance to discomfort” and inducing a positive bias in information processing; and (c) pro-social behavior by reducing “social fear” (De Jongh et al., 2008). Pharmacological neural or cognitive enhancement, indeed, has been defined as “the amplification or extension of the central capacities of the mind through the improvement or augmentation of internal and external information processing systems” (Sandberg and Savulescu, 2011).

According to Earp (2018), spiritual training, such as meditation, and in some cases, religion, can produce the same moral enhancement effects as the use of psychedelic drugs limited to an analogous environment. Furthermore, the concept of “moral improvement” itself is questioned by the author, as in spiritual, religious and mystical experiences, “moral improvement” is associated with affectivity, emotionality, empathy and cooperation rather than rational cognitive skills (Earp, 2018). For Earp, agency moral neurostimulation would be a subcategory of bioimprovement, with effects that would cause lasting changes in a moral agent, achieved (at least in part) through direct interventions in the Central Nervous System (CNS). In this case, some authors consider “moral neuroenhancement” or “moral bioenhancement” these kind of “technological” improvements (see Lavazza and Reichlin, 2019).

Ienca (2018) divides nootropics into three main families: neurochemical suppliers, nerve growth enhancers and antioxidants and neuroprotectives. Their actions on the CNS are diverse: they can alter the availability of the brain's supply of neurochemicals, such as neurotransmitters, hormones, and enzymes; or they can directly stimulate nerve growth; or, again, they can increase the brain's oxygen supply. The first category would include most of the nootropics targeted by scholars when they debate the use and abuse of PCEs. For example, one of the most widely discussed examples of the practice of bioenhancement is the use of psychostimulants such as amphetamine (Adderall), methylphenidate (Ritalin), and modafinil (Provigil).The universal use of these drugs by healthy people who use them to enhance their cognitive abilities is conceptualized as a PCE (Mohamed, 2015). There is sufficient evidence of the use of psychostimulant drugs by healthy people for cognitive improvement and to increase academic performance (Mohamed, 2015; Marraccini et al., 2016). In recent decades, trends have shown a strong positive attitude toward the use of these substances in the general population (Farah et al., 2004; Hall and Lucke, 2010; Husain and Mehta, 2011; Maier et al., 2018), with Modafinil being the most popular amongst the cognitive neuroenhancing drugs.

Pharmacological enhancement is the most massive and low-cost recreational neuroenhancement compared to the ones presented above, due to the technical simplicity of the drugs compared to other types of enhancement, with apparently no undesirable side effects, except for the risk of addiction and the prevalence of Alzheimer's disease, which some authors suggest could be triggered in individuals with a genetic predisposition (Bostrom and Roache, 2011). Despite the efforts made to propose categorisations of bio- or neuroenhancers, there is no agreement on whether pharmacological neuroenhancement is an activity distinct from other human activities aimed at improving “normal” functioning (and therefore deserving of specific regulation) or, as suggested by other authors such as Ienca, 2018, nootropic-induced enhancement should be considered in continuity with other non-pharmacological activities through which “our uniquely innovative species tries to improve itself” (Greely et al., 2008; Ienca, 2018). In this view, neuroenhancement would be more like a continuum, without a dichotomy between nootropics and non-nootropic enhancers.

Due to the above, it might be discussed if these “smart drugs” or “neuroenhancement drugs” (see Bublitz, 2016) fall by definition amongst the neurotechnologies addressed in the discussions about cognitive augmentation, and if, therefore, they should or not be considered part of the emerging network of neurorights proposed by Yuste et al. (2017).

## 4. The problem of equitable access to (recreational) neurotechnologies

The neuroright to cognitive enhancement could be considered more than a neuroright itself, a “normative ethical corollary” as Ienca, 2021b points out, since equitable access to cognitive improvement presupposes a “prerequisite to cognitive liberty”. Indeed, it is not possible to speak of cognitive liberty without considering the possibility of choosing to improve oneself, and if this lack is due to problems of equal access and/or knowledge of the said neurotechnologies, it seems an obvious contradiction. Moreover, equitable access should be ensured for all types of technology. Only by ensuring equity in knowledge, access and distribution of common goods we can guarantee the neuroright to cognitive freedom, and also dispel the dystopian fears of a social class of “enhanced humans”, a kind of neurocognitively enhanced elite able to control and exploit lower classes with normal or reduced cognitive abilities.

On the other hand, it could be observed that in the Chilean legislative project and the subsequent discussions did not address the important question of whether the enhancement of cognitive abilities really means “improving” the human being in a broader sense. It is clear that cognitive enhancement is more of an elective desire and wish than a basic need to be satisfied by the state, industry, commerce, and ultimately, the consumer and/or user of enhancement neurotechnologies.

Diego and Luisa Borbón-Rodríguez, in their critique of neurorights (Borbón and Borbón, 2021), including the right to cognitive enhancement and its equitable access (Borbón Rodríguez et al., 2020), point out that the boundaries between enhancement and transhumanism can become quite blurred, moving from a therapeutic medicine to a “medicine of desire” (Mainetti, 2008). This could even become a kind of contradiction in terms of free will, since the choice to be “improved” or enhanced would not really be an option for people. Social pressure would simply ensure this prerogative. “*The foregoing enters in contradiction with the proposed neuroright to free will in the sense that people would not be giving consent free of vices but falling in front of the new social norms created with this new right”* (Borbón Rodríguez et al., 2020). Ultimately, if the benefits of neural enhancement are provided by the state, they may overshadow other (more compelling) benefits, such as the therapeutic improvements themselves. Indeed, some authors have referred to neural enhancement as “Botox for the brain” (De Jongh et al., 2008), putting cognitive enhancement on the same level as aesthetic surgery. One might wonder whether the State should be in charge of our sumptuary choices or whether the person should be autonomously responsible for them. In this sense, and from a legal point of view, if the neurotechnologies used for cognitive enhancement turn out not to be harmless, who is responsible if the users' choice to use them might cause them mental or intellectual disability? In that case, should the state take full (or partial) responsibility for that disability, or should those who choose cognitively enhanceement, despite warnings of the risks, take out an insurance to cover the possible damages of their sumptuary choices, so to speak? Another open question, therefore, is whether cognitive improvement practices actually creates additional justice-related problems. In this sense, a specific regulation of the practice of pharmacological cognitive enhancement is advisable.

As a modern society (on the verge of Society 5.0; Deguchi et al., 2020), we cannot ignore or underestimate possible health risks due to the lack of empirical studies on how adaptive these changes we are facing will be and how profound they may be in our species: will they change human nature or represent an “event horizon” in the evolutionary process?

As discussed above, another consequence of the use and misuse of these neuroenhancing drugs could be the exacerbation of (already existing) social inequalities, in a future scenario in which those who are best placed in the consumer society will have easy access to these neurotechnologies, to the detriment of those who will not be able to do so, either because they don't know about them (knowledge asymmetry) or because they don't have the means to acquire them (access asymmetry). The consequences could be that these neuroenhancing drugs become part of the medicine of desire, or of the luxury goods that only the elite can consume. Indeed, some philosophers and legal scholars argue that what is unequally distributed is not neuromedicine in particular, but agency, which essentially implies autonomy and responsibility (Loewe, 2017).

## 5. Discussion and conclusions

There is currently no entitlement to cognitive enhancement or neural augmentation in the world. However, there is a proposal for fair or equitable access to cognitive enhancement by Yuste et al. (2017). This idea has been included in the Chilean draft law, colloquially called “neurorights”, but there is no specific mention about the right of equitable distribution of enhancing technologies in the articles regulating neurotechnologies. The right to fair access to mental augmentation “*seems to be a prerequisite for cognitive liberty in the positive sense”*, as Ienca points out.

Obviously, the difficulties in implementing this right go beyond the merely regulatory ones, since it requires first of all an agreement on the very concept of “enhancement”, and also requires major debates on who should have access to the different types of cognitive enhancement, not only from a therapeutic perspective, but also from a recreational or commercial one. This last point is the most controversial, because first of all, all stakeholders need to agree on the role of the state in non-therapeutic “enhancements” and the benefits to be conferred on each individual subject. Should the state guarantee those enhancements that allow healthy individuals to be “above normal”? Or should it only subsidize those who are below a threshold of “normality”? And how is “normality” defined? Or, again, should it only fund citizens who want to improve themselves by funding their sumptuary choices? If recreational users later become addicted or suffer from Alzheimer's disease as a result of using neuroenhancers, should the state support the future disabilities that will result, or will it be compulsory to take out insurance to cover the possible risks of commercial use?

These are the questions that must be discussed in order to reach a consensus on appropriate regulation, because it may be that the regulation of neurorights does not cover these necessary issues, but could be the first step in further discussion of a specific regulation. The truth is that before any state obligations for the betterment of citizens can be established, much more thought and debate is needed. What we suggest is that cognitive enhancement should take into account pharmacological enhancement, which is not only a type of non-invasive neurotechnology, but is also much more accessible and widespread, at least according to the statistics on the practice of neuroenhancement. Its silent expansion is taking place in the absence of regulation that incorporates ethical and neuroethical guidelines consistent with a debate based on human freedom, autonomy and dignity. Moreover, it is unclear whether “equity” refers only to access to information about neuroenhancers, or to possible financial subsidies to enable all kinds of people who want to improve themselves to have access to these products; or whether, on the other hand, it refers to the availability of PCEs to those who need to improve themselves, either because they have specific mental conditions that can be improved by NE, or because they perform specific jobs that require enhanced performance, for example, healthcare workers, surgeons, pilots, or those who work night shifts. In short, pharmacological cognitive neuroenhancement still needs a lot of discussion to focus on its contribution to society.

In the case of Chile, the proposed regulation could represent a step forward, as recreational and commercial neurotechnologies will adopt the health regulation in a complementary or residual way, in the absence of a specific law. In this sense, neuroenhancement devices for commercial use will be regulated by the medical model, just like any neurotechnological device for therapeutic purposes (Cornejo Plaza, 2021b). Currently, the draft law, Bulletin 13.828-19 (in progress), defines neurotechnologies without specifying whether or not they include pharmacological neuroenhancers. Given this omission, we believe it is important to clarify in the text whether or not PCEs are included in the right to enhancement. In this case, two scenarios could arise: a) if PCEs are excluded from the definition of neurotechnologies, since the practice of PNE is currently unrecognized and unsanctioned according to the legislation on pharmaceuticals, we propose to start working on a specific regulation that could improve the current legislation on drugs; b) if they are included, doctrine and jurisprudence could develop a position consistent with the neuroright to cognitive enhancement, supported by the Political Constitution of the Republic, which explicitly enshrines PCE (and this could have the consequences already discussed above). The problem with not specifying whether they are included or not is that it leaves room for interpretation.

With regard to legislation on pharmaceuticals, we believe that the use of neuroenhancers should be specifically reconsidered, distinguishing their use according to the therapeutic or non-therapeutic use sought by the individual, also taking into account the activities and ages of the individuals. We are, indeed, completely unaware of the potential damage that PCEs can cause to the developing brain, because we do not have conclusive studies on smart drugs usage in children or early adolescents. Taking into account the possible adverse or side-effects of the use of such drugs, as discussed above, and the concrete possibility of developing a psychological dependence on them, it would seem a good idea to take responsibility for the issue and eventually to educate the public about the consequences of the misuse of these recreational pharmacological enhancers.

## Author contributions

All authors listed have made a substantial, direct, and intellectual contribution to the work and approved it for publication.
